# Exploring locality sensitive hashing as a blocking method for large-scale administrative datasets

**DOI:** 10.23889/ijpds.v8i2.2318

**Published:** 2023-09-14

**Authors:** Leah Quinn, Rachel Shipsey

**Affiliations:** 1 Office for National Statistics, Portsmouth, United Kingdom

## Objectives

Linking large-scale datasets is challenging due to the computational power required. This research explores using Locality-Sensitive-Hashing (LSH) as a blocking method to reduce the computational complexity when linking large administrative datasets. LSH hashes similar data into ‘buckets’, thus reducing the search space and processing power required to find links.

## Methods

A gold-standard linked dataset was used during method development. Test datasets were made using samples of gold-standard matches and non-matches, then blocked using LSH.

Various LSH parameters including shingle length, signature length, band size and number of matching bands were tested. Precision and recall were used to find optimal parameters for identifying good candidate pairs, with 100% recall and >20% precision being desirable.

Alternative formats for date of birth, postcode and gender variables were tested, with additional characters used to simulate agreement weighting.

## Results

Results as of spring 2023 are promising, with the caveat that currently only small datasets have been tested. The LSH method with optimal parameters creates ~9,000 candidate pairs whilst maintaining recall of 100% (i.e., all true matches are included in the candidate pairs) and precision of 27.6%. In contrast, our traditional deterministic blocking method using the same variables creates ~70,000 candidate pairs, and a cartesian product creates over 23.4 million candidate pairs. We have therefore shown that LSH can be used to create a significant reduction in the search-space size.

Furthermore, the method easily handles alternative names, postcodes, etc. that may be present in longitudinal data or composite datasets, with no need to account for different possible combinations of variables.

## Conclusion

Current research has shown that LSH can be used to drastically reduce the search space when blocking for data linkage. Using variable formatting to prioritise agreement for specific sections e.g., of postcode, has overcome a potential downside of LSH. Further research on variable formatting, parameter optimisation and testing of the method at scale is ongoing.

